# Determinants of frequent attendance in Danish general practice: a cohort-based cross-sectional study

**DOI:** 10.1186/s12875-016-0412-4

**Published:** 2016-01-28

**Authors:** Jeanette Therming Jørgensen, John Sahl Andersen, Anne Tjønneland, Zorana Jovanovic Andersen

**Affiliations:** Department of Public Health, Centre for Epidemiology and Screening, University of Copenhagen, Copenhagen, Denmark; Department of Public Health, Section of General Practice, University of Copenhagen, Copenhagen, Denmark; Danish Centre for Cancer Research, Danish Cancer Society, Copenhagen, Denmark

**Keywords:** Frequent attendance, General practice, Gender, Lifestyle, Unemployment, Cohort

## Abstract

**Background:**

Previous studies addressing determinants of frequent attendance have mainly focused on socio-demographic, psychosocial and medical factors, and few had data on lifestyle and gender-specific factors. This study aims to describe determinants of general practice frequent attendance in Danish adult population, by examining lifestyle, socio-demographic, medical and gender-specific factors.

**Method:**

For 54,849 participants of the Danish Diet, Cancer and Health cohort (50–65 year old) we obtained data on visits to general practitioner (GP) from the Danish National Health Service Register at cohort baseline (1993–97), when information on medical conditions and lifestyle, socio-demographic and gender-specific factors was collected by questionnaire. Logistic regression was used to identify determinants of frequent attendance, defined as top 10 % GP users at the year of recruitment into the cohort (baseline) in the period between 1993 and 1997.

**Results:**

Frequent attenders accounted for 40 % of all face-to-face GP consultations with a mean 12 visits/year. Women were more likely to be frequent attenders, in crude (Odds ratio: 1.95; 95 % Confidence Interval: 1.85–2.06) and fully adjusted (1.26; 1.09–1.47) model. In a fully adjusted model, strongest determinants of frequent attendance were pre-existing medical conditions, with hypertension (2.58; 2.42–2.75), diabetes (2.24; 1.94–2.59), and mental illness (2.29; 2.09–2.52) more than doubling the odds of being FA. High education (0.63; 0.57–0.69, >4 years higher education vs. no vocational training) and employment (0.61; 0.57–0.65) were inversely associated with frequent attendance. Finally, obesity (1.54; 1.14–2.08), smoking (1.21; 1.12–1.30, current vs. never), physical activity (0.84; 0.80–89), alcohol consumption (0.83; 0.78–0.87 above vs. below recommended level), and hormone therapy in women (1.52; 1.42–1.63) were all significant determinants of frequent attendance.

**Conclusions:**

In addition to pre-existing medical conditions, gender, socio-demographic and gender-specific factors, lifestyle (obesity, smoking, exercise and alcohol use) is also an independent determinant of frequent attendance at general practitioner.

## Background

Frequent attendance in general practice and the management hereof has been widely discussed in previous literature. Results from systematic reviews reveal that frequent attendance, typically defined as the top 10^th^ percentile of general practitioner (GP) attenders [1], is associated with social factors (unemployment, divorce, low education and social support), psychological distress, and physical health [2, 3]. However, results from a Danish study addressing the influence of social factors, suggests that male frequent attendance is associated with living alone, being out of work or on disability pension, whereas female frequent attendance seems unaffected by social factors, when analysis is adjusted for physical and psychological health [4]. Few studies addressing determinants of frequent attendance included information on lifestyle factors, and majority of them based their results on a limited sample size [5, 6]. One study examined the effect of body mass index (BMI) on FA and reported that overweight and obesity’s effect on GP use in Denmark was found to vary across classes, and that overweight or obesity only affected GP use among frequent attenders (FAs) [7], suggesting variation in characteristics and underlying mechanism between GP use among FAs and the general population. Others addressed risk factors of persistent frequent attendance, and identified female gender, obesity (>30 kg/m^2^), former frequent attendance, a broad spectrum of psychosocial factors, alcohol abstinence, low patient satisfaction, medically unexplained physical symptoms, and chronic diseases as predictive risk factors for persistent frequent attendance [5, 8, 9]. Further, one study estimated lifestyle factors’ effect on higher than average attendance (top 25^th^ percentile attenders), where increased alcohol consumption was negatively associated with higher than average attendance, whereas smoking and physical activity had no effect [6].

In this study, we examine determinants of frequent attendance in Danish men and women aged 50 to 65 years, and estimate effect of lifestyle factors when analyses are adjusting for gender, age, marital, occupational and educational status, urbanization, pre-existing diseases (somatic and mental), history of cancer in the family, and female-specific factors (parity, use of postmenopausal hormone therapy (HT) and contraceptives).

## Methods

### Design and setting

We have linked data on 57,053 participants of the Danish Diet, Cancer and Health (DCH) cohort to the Danish National Health Service Register (NHSR) to obtain data on visits to GPs. This study was conducted as a cohort-based cross-sectional study, where information on GP visits and confounder information were collected within the same year, at cohort baseline in 1993–97.

### Danish National Health Service Register

NHSR is a nationwide register containing information on all contacts within primary health care in Denmark [10]. The register was established for administrative purposes in 1984, and data has been available for research purposes since 1990 [10, 11]. Due to administrative purpose of the Danish National Health Service Register, services provided in primary health care are reported and reimbursed on a weekly basis, meaning that the total number of visits per week for a given person can be obtained from the register. In addition to citizen-related data, NHSR contains information on the health care provider and the type of service provided (e.g., telephone consultation, home-visit, face-to-face consultation, preventive consultation). Reasons for encounter or information on specific health problems is only available through NHSR to a limited extent, in terms of service codes (e.g., prescription renewal, additional service codes), and no diagnoses are available. GP visits in this study were defined as sum of all face-to-face contacts at the year of cohort baseline (1993–97), including consultations at the GPs office and home-visits during opening hours in general practice, while telephone consultations and prescription renewals were excluded. Frequent attendance was defined as the top 10^th^ percentile of GP users. Further, information on cohort members’ visit to psychologist and psychiatrist within the Danish primary health sector was obtained from NHSR and used as an indicator of mental health status.

### Danish Diet, Cancer, and Health Cohort

The DCH cohort, described in detail elsewhere [12], is part of the EPIC cohorts and used widely for the research into lifestyle factors, with focus on diet and the risk of cancer and other chronic diseases. During 1993–97 a total of 160,725 individuals, aged between 50 and 64, born in Denmark, living in Copenhagen or Aarhus, and with no previous records in the Danish Cancer Registry [13], were invited to participate in the DCH cohort study. A total of 57,053 individuals participated in the study. Cohort participation involved answering comprehensive questionnaires and interviews concerning dietary intake and lifestyle factors that are known potential risk factors in the development of cancer. Additionally, anthropometric measurements were taken during a physical examination and various biological materials were collected. The following potential determinants of FA were obtained from the DCH cohort: gender, age, BMI, alcohol consumption, smoking status, leisure time physical activity, marital status, occupational status, educational status, urbanization, pre-existing diseases, history of cancer in the family, and gender-specific information including number of pregnancies, use of HT and OC. Age refers to the participants’ age at the date of the physical examination, and apart from age, gender, BMI, and urbanization, all other variables collected from the DCH cohort are self-reported. Urbanization was dichotomized into urban (Copenhagen, Frederiksberg or Aarhus municipality) and suburban (remaining suburban municipalities around Copenhagen and Aarhus). Further, participants were classified as either employed or unemployed. The self-reported daily alcohol intake in grams was dichotomized (below and above the recommended limit) according to Danish Health and Medicines Authority’s recommendation on weekly alcohol consumption at the time of cohort baseline, corresponding to 168 grams alcohol for females and 252 grams for males. Prevalence of pre-existing diseases was estimated based on participants self-reporting either being diagnosed with the given disease or self-reported use of medication to treat the disease.

### Statistical analysis

Logistic Regression model was used for exploring effects of before-mentioned covariates on FA. We examined how the probability of being classified as FA was related to the cohort participants’ lifestyle, socio-demography, medical conditions and gender-specific characteristics. Two models will be fit: 1) Model 1, a crude model, adjusted for age and gender and one other variable at the time, 2) Model 2, a fully adjusted model. Additionally, the two models were fit for men and women separately, and interaction terms between gender and all other covariate were introduced in fully adjusted model one at a time, to test potential effect modification. Results are presented as Odds Ratio (OR) with 95 % confidence intervals (95 % CI). Logistic regression models were performed using PROC LOGISTIC procedures in SAS 9.3. Relevant Danish ethical committees and Danish Data Protection Agency have approved the study (J.nr. 2013-41-1600), and written informed content was provided by all participants at recruitment.

## Results

Of the 57,053 DCH cohort participants, 571 were excluded due to cancer diagnosis prior to cohort baseline and 1,633 due to missing values on one or more covariates of interest, leaving 54,849 cohort members for analyses of whom 28,643 (52.2 %) were women. When applying the top 10^th^ percentile frequent attendance limit, cohort members with ≥ 8 GP visits at the year of baseline were classified as FAs and members with < 8 annual contacts as non-FAs. A total of 188,709 GP visits were registered in NHSR for 54,849 cohort members at cohort baseline year (1993–97) and FA visits accounted for 39.6 % of these. FAs consulted their GP on average 12.0 (standard deviation: 7.1) times a year, while non-FAs had 2.3 (2.0) annual GP visits. Of the cohort, 6,226 (11.3 %) were classified as FAs and of these 4,143 (66.5 %) were women (Table 1). Among non-FAs, women had 2.7 (2.0) annual GP visits, while men had 1.9 (1.9). In addition, gender did not seem to influence the number of GP visits among FAs, since male FAs had 12.1 (7.9) annual visits and female had 12.0 (6.7). Among non-FAs, GP visits were almost equally distributed across different categories of BMI, alcohol consumption, smoking status and leisure time physical activity. The same pattern applies for FAs, except for BMI, where underweight individuals (BMI below 18.5 kg/m^2^) consulted their GP more often than individuals in other BMI classes (underweight, normal, overweight, obese; 15.6, 12.0, 11.8, 12.3). In a crude age-adjusted model, women had 1.95 (1.85–2.06) higher odds of being FAs than men (Table 2). This significant frequent attendance variation between genders persisted, but attenuated in a fully adjusted model (OR 1.26; 95 % confidence interval 1.09–1.47). In crude model, age was positively associated with frequent attendance, where odds of being FA was 28 % (1.28; 1.20–1.37) higher among cohort members aged between 55 to 59 year, and 54 % (1.54; 1.45–1.65) higher among those aged between 60 to 65, relative to cohort members in the age group of 50 to 54 years. This positive effect did, however, disappear in the fully adjusted model. Similarly, in crude model, the effect of high BMI was significant, with obese participants having 87 % (1.87; 1.40–2.51) higher odds of being FAs, which persisted but attenuated in fully adjusted model (1.54; 1.14–2.08). Participants drinking above the recommended weekly limit of alcohol had significantly 17 % lower odds of being FAs compared to those restricting their alcohol consumption to recommendations (0.83; 0.78–0.87). Smoking was associated with frequent attendance, with current (1.21; 1.13–.1.30) and previous (1.21; 1.12–1.30) smokers having higher odds of being FAs than never smokers. Physically active participants had lower odds of being FAs (0.84; 0.80–0.89). Married participants were less likely to be FAs (0.83; 0.73–0.94) as compared to unmarried participants. Occupational status was a strong determinant of frequent attendance, with employed participants being 39 % (0.61; 0.57–0.65) less likely to be FAs compared to those who were unemployed. Furthermore, an increase in educational level was negatively associated with odds of being FA. Pre-existing diseases were the strongest determinants of frequent attendance, with hypertension (2.58; 2.42–2.75), mental illness (2.29; 2.09–2.52) and diabetes (2.26; 1.96–2.61) as leading determinants, more than doubling the odds of being FA. No association was found among women’s use of OC and the likelihood of being FA, whereas current or previous users of postmenopausal HT had 52 % (1.58; 1.42–1.63) higher odds of being FA than never users. An increased odds of being FA was found among women who had one or more pregnancies (1.17; 1.02–1.34) compared to women with no previous history of pregnancy. Significant effect modification by gender was identified for a number of factors, including age, smoking, employment status, hypertension and diabetes (Table 3). The positive association between age, smoking, unemployment, hypertension and diabetes and frequent attendance was significantly higher in men than in women.

**Table 1:** Number of GP visits among non-FAs and FAs in DCH (*N* = 54,849) at baseline in 1993–97

	Non-FAs		FAs	
	*N* = 48,623		*N* = 6,226	
	*N*	Mean (std)	*N*	Mean (std)
Gender				
Male	24,123	1.95 (1.95)	2,083	12.1 (7.90)
Female	24,500	2.73 (2.05)	4,143	12.0 (6.67)
Age				
50–54	20,979	2.21 (1.99)	2,181	12.1 (7.14)
55–59	15,022	2.39 (2.06)	2,006	12.2 (8.05)
60–65	12,622	2.51 (2.09)	2,039	11.8 (6.00)
BMI				
Underweight (<18.5 kg/m^2^)	366	2.60 (2.06)	55	15.6 (15.0)
Normal weight (18.5–24.9 kg/m^2^)	21,438	2.26 (1.99)	2,153	12.0 (7.49)
Overweight (25–29.9 kg/m^2^)	20,301	2.31 (2.04)	2,582	11.8 (6.42)
Obese (≥30 kg/m^2^)	6,518	2.70 (2.16)	1,436	12.3 (7.19)
Alcohol consumption				
Below the recommended weekly limit	28,518	2.38 (2.06)	4,052	12.4 (7.94)
Above the recommended weekly limit	20,105	2.29 (2.02)	2,174	11.4 (5.17)
Smoking status				
Never	17,299	2.34 (2.03)	2,039	11.8 (6.77)
Previous	13,992	2.37 (2.05)	1,834	11.9 (5.93)
Current	17,332	2.32 (2.04)	2,353	12.3 (8.15)
Physical activity				
No leisure time physical activity	21,982	2.37 (2.08)	3,242	12.2 (7.59)
Physically active in leisure time	26,641	2.32 (2.01)	2,984	11.8 (6.54)
Marital status				
Unmarried	2,880	2.18 (2.03)	393	12.4 (7.60)
Divorced	7,913	2.50 (2.08)	1,331	12.4 (8.03)
Widow/widower	2,562	2.69 (2.10)	455	12.3 (8.20)
Married	35,268	2.30 (2.02)	4,047	11.8 (6.58)
Occupational status				
Unemployed	9,739	2.82 (2.15)	2,330	12.6 (8.07)
Employed	38,884	2.22 (1.99)	3,896	11.7 (6.45)
Educational status				
No vocational training	6,638	2.72 (2.12)	1,466	12.4 (7.11)
Higher education, <3 years	10,878	2.56 (2.07)	1,569	11.7 (5.39)
Higher education, 3–4 years	19,845	2.32 (2.02)	2,278	12.0 (8.26)
Higher education, >4 years	11,262	1.96 (1.93)	913	11.8 (6.54)
Urbanization				
Suburban	21,695	2.33 (2.03)	2,753	11.9 (7.12)
Urban	26,928	2.36 (2.05)	3,473	12.1 (7.10)
Medical conditions				
Heart attack	877	3.22 (2.13)	244	13.3 (10.3)
High cholesterol	3,284	3.02 (2.11)	801	12.1 (5.87)
Angina pectoris	1,226	3.26 (2.13)	423	12.6 (8.02)
Stroke	536	3.32 (2.13)	174	12.5 (5.84)
Hypertension	6,758	3.37 (2.10)	2,152	12.3 (7.58)
Diabetes	800	3.30 (2.20)	333	13.7 (10.6)
Gallstones	2,065	2.95 (2.13)	559	12.4 (6.92)
Intestinal polyps	1,619	2.70 (2.10)	300	12.4 (6.53)
Mental illness	2,252	3.23 (2.12)	745	13.8 (10.9)
History of cancer in the family	23,187	2.39 (2.06)	3,087	12.0 (6.48)
Oral contraceptives (OC) use				
Never	34,292	2.17 (2.01)	3,873	12.1 (7.27)
Previous or current	14,331	2.76 (2.05)	2,353	11.9 (6.84)
Hormone therapy (HT)				
Never	38,279	2.15 (2.00)	3,984	11.9 (6.99)
Previous or current user	10,344	3.05 (2.05)	2,242	12.3 (7.31)
Number of pregnancies				
0	26,293	2.00 (1.96)	2,392	12.1 (7.69)
1 or more	22,330	2.75 (2.06)	3,834	12.0 (6.72)

*FAs* frequent attenders, *BMI* body mass index

**Table 2:** Determinants of frequent attendance among men and women in the DCH cohort (*N* = 54,849)

	Frequent attendance	
	Crude model^a^	Adjusted model^b^
	OR (95 % CI)	OR (95 % CI)
Gender		
Male (*n* = 26,206)	1.00	1.00
Female (*n* = 28,643)	1.95 (1.85–2.06)	1.26 (1.09–1.47)
Age		
50–54	1.00	1.00
55–59	1.28 (1.20–1.37)	1.07 (1.00–1.14)
60–65	1.54 (1.45–1.65)	1.04 (0.97–1.12)
BMI		
Underweight (<18.5 kg/m^2^)	1.00	1.00
Normal weight (18.5–24.9 kg/m^2^)	0.79 (0.60–1.06)	0.92 (0.68–1.24)
Overweight (25–29.9 kg/m^2^)	1.12 (0.84–1.49)	1.16 (0.86–1.57)
Obese (≥30 kg/m^2^)	1.87 (1.40–2.51)	1.54 (1.14–2.08)
Alcohol consumption		
Below the recommended weekly limit	1.00	1.00
Above the recommended weekly limit	0.79 (0.74–0.83)	0.83 (0.78–0.87)
Smoking status		
Never	1.00	1.00
Previous	1.27 (1.18–1.36)	1.21 (1.12–1.30)
Current	1.29 (1.21–1.38)	1.21 (1.13–1.30)
Physical activity		
No leisure time physical activity	1.00	1.00
Physically active in leisure time	0.71 (0.68–0.75)	0.84 (0.80–0.89)
Marital status		
Unmarried	1.00	1.00
Divorced	1.18 (1.05–1.34)	1.03 (0.90–1.17)
Widow/widower	1.00 (0.86–1.15)	0.87 (0.75–1.03)
Married	0.85 (0.76–0.95)	0.83 (0.73–0.94)
Occupational status		
Unemployed	1.00	1.00
Employed	0.48 (0.45–0.51)	0.61 (0.57–0.65)
Educational status		
No vocational training	1.00	1.00
Higher education, <3 years	0.65 (0.60–0.70)	0.77 (0.71–0.84)
Higher education, 3–4 years	0.59 (0.55–0.63)	0.72 (0.66–0.78)
Higher education, >4 years	0.48 (0.44–0.52)	0.63 (0.57–0.69)
Urbanization		
Suburban	1.00	1.00
Urban	1.02 (0.97–1.08)	0.95 (0.90–1.01)
Medical conditions		
Heart attack	2.62 (2.26–3.04)	1.15 (0.97–1.37)
High cholesterol	2.11 (1.94–2.29)	1.38 (1.25–1.51)
Angina pectoris	2.98 (2.65–3.34)	1.62 (1.42–1.86)
Stroke	2.67 (2.24–3.18)	1.47 (1.22–1.78)
Hypertension	3.15 (2.97–3.34)	2.58 (2.42–2.75)
Diabetes	3.68 (3.22–4.20)	2.24 (1.94–2.59)
Gallstones	1.81 (1.64–2.00)	1.41 (1.27–1.56)
Intestinal polyps	1.50 (1.32–1.70)	1.26 (1.10–1.45)
Mental illness	2.60 (2.38–2.85)	2.29 (2.09–2.52)
History of cancer in the family	1.04 (0.99–1.10)	1.04 (0.99–1.10)
Oral contraceptives (OC) use		
Never	1.00	1.00
Previous or current	1.02 (0.96–1.09)	1.03 (0.95–1.10)
Hormone therapy (HT)		
Never	1.00	1.00
Previous or current user	1.57 (1.47–1.68)	1.52 (1.42–1.63)
Number of pregnancies		
0	1.00	1.00
1 or more	1.23 (1.09–1.39)	1.17 (1.02–1.34)

^a^Adjusted for gender and age

^b^Fully adjusted model for all covariates listed in Table 2

**Table 3:** Determinants of frequent attendance in the DCH cohort (*N* = 54,849) by gender

	Frequent attendance				
	Female (*N* = 28,643)		Male (*N* = 26,206)		
	Crude^a^ model	Adjusted^b^ model	Crude^a^ model	Adjusted^b^ model	*P*-value^c^
	OR (95 % CI)	OR (95 % CI)	OR (95 % CI)	OR (95 % CI)	
Age					
50–54	1.00	1.00	1.00	1.00	-
55–59	1.19 (1.10–1.29)	0.96 (0.88–1.04)	1.49 (1.33–1.66)	1.31 (1.16–1.47)	< .0001
60–65	1.34 (1.24–1.46)	0.89 (0.81–0.98)	1.99 (1.78–2.22)	1.37 (1.21–1.55)	< .0001
BMI					
Underweight (<18.5 kg/m^2^)	1.00	1.00	1.00	1.00	-
Normal weight (18.5–24.9 kg/m^2^)	0.87 (0.64–1.19)	0.98 (0.71–1.35)	0.39 (0.19–0.80)	0.66 (0.31–1.40)	0.1169
Overweight (25–29.9 kg/m^2^)	1.30 (0.95–1.78)	1.30 (0.94–1.80)	0.53 (0.26–1.07)	0.56 (0.26–1.19)	0.0819
Obese (≥30 kg/m^2^)	1.94 (1.41–2.66)	1.60 (1.15–2.23)	1.02 (0.50–2.07)	0.97 (0.45–2.05)	0.2235
Alcohol consumption					
Below the recommended weekly limit	1.00	1.00	1.00	1.00	-
Above the recommended weekly limit	0.71 (0.67–0.77)	0.79 (0.73–0.85)	0.93 (0.85–1.02)	0.88 (0.80–0.97)	0.0519
Smoking status					
Never	1.00	1.00	1.00	1.00	-
Previous	1.19 (1.09–1.29)	1.16 (1.06–1.26)	1.44 (1.27–1.63)	1.33 (1.16–1.51)	0.0262
Current	1.27 (1.18–1.37)	1.19 (1.09–1.29)	1.39 (1.23–1.63)	1.29 (1.14–1.48)	0.1491
Physical activity					
No leisure time physical activity	1.00	1.00	1.00	1.00	-
Physically active in leisure time	0.74 (0.70–0.79)	0.86 (0.80–0.93)	0.67 (0.61–0.74)	0.85 (0.77–0.94)	0.2173
Marital status					
Unmarried	1.00	1.00	1.00	1.00	-
Divorced	1.29 (1.11–1.51)	1.05 (0.88–1.25)	1.02 (0.84–1.24)	1.01 (0.82–1.25)	0.9283
Widow/widower	1.14 (0.96–1.36)	0.94 (0.78–1.15)	0.86 (0.63–1.17)	0.87 (0.62–1.21)	0.7943
Married	0.99 (0.85–1.14)	0.89 (0.76–1.05)	0.65 (0.54–0.77)	0.76 (0.63–0.91)	0.2726
Occupational status					
Unemployed	1.00	1.00	1.00	1.00	-
Employed	0.52 (0.48–0.56)	0.65 (0.61–0.71)	0.40 (0.35–0.44)	0.53 (0.48–0.60)	< .0001
Educational status					
No vocational training	1.00	1.00	1.00	1.00	-
Higher education. <3 years	0.65 (0.59–0.71)	0.76 (0.69–0.84)	0.64 (0.54–0.75)	0.80 (0.67–0.95)	0.9217
Higher education. 3–4 years	0.59 (0.54–0.64)	0.71 (0.65–0.78)	0.58 (0.51–0.67)	0.71 (0.61–0.82)	0.5737
Higher education. >4 years	0.47 (0.41–0.53)	0.63 (0.55–0.73)	0.48 (0.41–0.55)	0.62 (0.53–0.72)	0.4581
Urbanization					
Suburban	1.00	1.00	1.00	1.00	-
Urban	1.00 (0.94–1.07)	0.95 (0.89–1.02)	1.07 (0.97–1.17)	0.94 (0.86–1.04)	0.9194
Medical conditions					
Heart attack	2.64 (2.01–3.47)	1.15 (0.85–1.57)	2.54 (2.13–3.03)	1.07 (0.87–1.32)	0.5512
High cholesterol	1.95 (1.74–2.18)	1.37 (1.211–1.55)	2.35 (2.07–2.65)	1.39 (1.20–1.59)	0.1549
Angina pectoris	2.94 (2.48–3.48)	1.65 (1.36–1.99)	2.96 (2.52–3.47)	1.53 (1.27–1.86)	0.4896
Stroke	2.67 (2.07–3.44)	1.66 (1.26–2.18)	2.57 (2.01–3.28)	1.20 (0.92–1.56)	0.6247
Hypertension	2.70 (2.50–2.90)	2.27 (2.09–2.46)	4.13 (3.75–4.55)	3.25 (2.92–3.60)	<.0001
Diabetes	3.05 (2.49–3.74)	1.85 (1.49–2.31)	4.20 (3.52–5.00)	2.48 (2.05–3.00)	0.0032
Gallstones	1.87 (1.68–2.08)	1.45 (1.29–1.63)	1.64 (1.28–2.10)	1.30 (1.00–1.69)	0.8436
Intestinal polyps	1.51 (1.27–1.78)	1.20 (1.00–1.44)	1.47 (1.21–1.79)	1.36 (1.10–1.67)	0.2483
Mental illness	2.51 (2.26–2.78)	2.23 (2.00–2.49)	2.88 (2.42–3.43)	2.48 (2.06–2.99)	0.4134
History of cancer in the family	1.06 (0.99–1.13)	1.05 (0.98–1.12)	1.01 (0.92–1.11)	1.03 (0.94–1.13)	0.7695
Oral contraceptives (OC) use					
Never	1.00	1.00	-	-	-
Previous or current	0.99 (0.93–1.06)	0.99 (0.92–1.06)			
Hormone therapy (HT)					
Never	1.00	1.00	-	-	-
Previous or current user	1.59 (1.49–1.70)	1.54 (1.44–1.65)			
No. of pregnancies					
0	1.00	1.00	-	-	-
1	1.21 (1.04–1.42)	1.15 (0.97–1.36)			
2	1.13 (0.99–1.29)	1.13 (0.97–1.32)			
3	1.16 (1.01–1.33)	1.11 (0.95–1.30)			
4	1.30 (1.12–1.50)	1.14 (0.96–1.35)			
5	1.52 (1.28–1.80)	1.25 (1.03–1.51)			
6	1.54 (1.23–1.92)	1.18 (0.92–1.50)			
7	1.55 (1.11–2.17)	1.11 (0.77–1.57)			
8	2.12 (1.44–3.12)	1.51 (0.99–2.29)			

^a^Adjusted for age

^b^Fully adjusted model

^c^ *P*-value for interaction between gender and given covariate

## Discussion

### Summary of main findings

This study yielded three major findings: 1) frequent attendance defined as top 10^th^ percentile GP users, accounted for 39.6 % of all GP visits, 2) lifestyle factors including BMI, smoking, alcohol consumption and leisure time physical activity, explained some of the variation in GP use between FAs and non-FAs, however, 3) female gender, unemployment, low level of education, pre-existing medical conditions, and use of HT were identified as leading determinants of frequent attendance.

### Strengths and limitations of the study

Strength of this study is a large Danish cohort consisting of 57,053 participants recruited from general population, with high quality data on lifestyle, education, diseases, and measured height and weight. NHSR is generally considered to be of high validity for several reasons, including its administrative purpose, and the GPs financial dependency upon it [11]. There are several limitations to this study. Until January 1996, services provided to children under the age of 16 were reported on parents’ person identification number, and such records were marked with a special notation in the register, indicating them to be children’s record. These records were removed from the data in this study. However, inconsistencies in these records have been suggested, since providers often forgot to mark children’s records, due to equal fees for services provided to children and adults [11]. Inconsistencies may have led to an overestimation of female records in the register before 1996, since it has been suggested that women more often take their children to GP compared to men [11]. However, this inconsistency is considered as unlikely to have had influence on present results, when taking the DCH participant’s age at cohort enrolment (50–65 years) into account. All information on pre-existing diseases was self-reported, and thus, the actual number of pre-existing diseases is probably underestimated due to undiagnosed diseases or recall bias and leak of self-awareness and knowledge of one’s medical condition Furthermore, information on the severity of the diseases was unavailable. The variable on occupational status was constructed based on participants’ self-reported levels of physical activity at work, and dichotomized into unemployed and employed, and it was not possible to distinguish between individuals who were unemployed from those who were retired, on sick leave, early retired or on disability pension. Further, classification of participants with mental illness was based on contacts with psychologist or psychiatrist within the primary healthcare sector, and lack of information on participants’ mental hospital admissions or use of medication to treat mental conditions may have led to misclassification. Current promotion of a healthier lifestyle e.g., stress on prevention of lifestyle-related chronic diseases like diabetes and cardiovascular diseases and the introduction of smoking bans in 2007 may have had an impact on the lifestyle in the Danish population, and estimates based on 1993–97, may be slightly different from current tendencies. Lastly, others have pointed out the importance of distinguishing between temporary and persistent frequent attendance [14], and noted that interventions aimed at reducing unnecessary consultations in general practice should be targeted to the latter group of patients, since they carry the excessive economic burden in general practice. However, this study design did not allow us to discriminate between different types of frequent attendance.

### Comparison with existing literature

FAs consulted their GP on average 12.0 (7.1) times a year, while non-FAs had 2.3 (2.0) annual visits to GP, which is consistent with previous findings by Gupta and Greve who use the National Health Interview Survey from 2000 of Danish individuals aged 25–60 and report 13.2 (8.8) mean GP visits in FAs and 3.1 (3.0) visits in non-FAs [7]. Our results show an association between female gender and frequent attendance, and that FA visits accounted for 39.6 % of all GP visits, which supports previous evidence [2, 8], and further, analysis based on the general Danish adult population revealed that female have 18 % more face-to-face GP visits, compared to men (unpublished work). In a fully adjusted model, the effect of age was insignificant. However, when considering the effect of age separately for men and women, age was significantly modified by gender. Women aged 60–65 years had significantly smaller (0.89; 0.81–0.98) while men aged 60–65 had significantly higher odds (1.37; 1.21–1.55) of being FAs as compared to 50–54 old women and men, respectively. This variation may be explained by higher utilization related to menopause and menopausal symptoms (other than use of HT) among women in the age group of 50–59 years when most women experience onset of menopause. The number of GP contacts (consultations, home-visits and telephone consultations) has increased over the last few decades [15], meaning that estimates on average GP visits reported in our paper may be lower than present values. However, Moth et al. reported that only minor changes in overall patterns of reasons for encounter were seen from 1993 to 2009, implying that mechanism influencing individuals to consult their GP would not differ substantially today [16].

#### Lifestyle factors

We found a significant association between obesity and frequent attendance, which is consistent with Gupta and Greve findings, however, unlike their results we did not find an association between overweight and frequent attendance [7]. Inverse association between alcohol consumption and frequent attendance reported in this study is similar to findings by Little et al. [6], but comparison must be done with caution, since their study examined higher than average attendance (top 25^th^ percentile). Little et al., also examined associations between other lifestyle factors and higher than average attendance, and found no association with smoking status or physical activity, in contrast to our results. No previous studies have estimated effect of alcohol use, smoking and physical activity on frequent attendance, so these novel results need to be reproduced.

#### Socio-demographic factors

Our findings of an association between unemployment and frequent attendance is consistent with existing evidence from a systematic literature review [2], but contradicts results from another study which reported that only male frequent attendance was associated with living alone, being out of work or on disability pension when controlling for physical and psychological health, whereas female frequent attendance was unaffected by social factors [4]. In our study frequent attendance was strongly associated with unemployment in both genders, although significantly stronger in males (0.53; 0.48–0.60) than females (0.65; 0.61–0.71). Similarly, we found significant associations between frequent attendance and educational level. With relation to marital status, no significant difference in the frequent attendance were found between female participants classified as married, unmarried, divorced or widowed, whereas married men had 24 % lower odds of being FAs compared to unmarried male participants.

#### Medical conditions

It is well known that the prevalence of chronic diseases and physical illness is higher among FAs [2], and results from present study supports this evidence, by showing that the existence of most diseases increased the odds of being FA significantly, with exception of individuals who had a heart attack prior to cohort baseline. However, the effect of a previous heart attack may be correlated with the presence of high cholesterol and hypertension, and possibly explaining why the effect disappears in the fully adjusted model. Further, the insignificant effect may be due to the fact that the majority of treatment regimens post heart attack are provided and managed by ambulatory care within the secondary health care sector.

#### Gender-specific factors

Strong gender difference observed in the crude model (1.95; 1.85–2.06) remained robust after adjusting for medical conditions, socio-economic and lifestyle factors, but attenuated the most when female reproductive factors were included in the model (results not shown). For women, use of HT was strongly associated with frequent attendance, with previous or current users having 54 % higher odds of being FAs, compared never users. Furthermore, women who reported having been pregnant one or more times had 17 % higher odds of being FAs than those with no history of pregnancies. Since records related to children were removed from the data and the study population was aged above 50 years, the influence of parity may seem strange, nevertheless the higher attendance rates among women who went through pregnancy may be explained by the fact that women who have had a pregnancy experience long-term consequences caused by vaginal delivery, e.g., problems related to incontinence, bladder infections or genital prolapse [17, 18].

## Conclusions

In this study we have shown that patients’ gender, medical (somatic and mental) conditions, socio-demography and gender-specific GP use do not solely explain variation in FA or non-FA characteristics. Additionally, patients’ lifestyle explains some of the variation between FAs and non-FAs, and future interventions within general practice, targeted to help patients with high attendance rates, and possibly reduce unnecessary use of GP, should take this into consideration.

## Abbreviations

GP: General practitioner
FAs: Frequent attenders
NHSR: National Health Service Register
DCH: Diet, Cancer and Health
HT: Hormone therapy
OC: Oral contraceptives
OR: Odds ratio
95 % CI: 95 % Confidence intervals
